# Higher risk of hepatocellular carcinoma in Hispanic patients with hepatitis C cirrhosis and metabolic risk factors

**DOI:** 10.1038/s41598-018-25533-2

**Published:** 2018-05-08

**Authors:** Alina Wong, An Le, Mei-Hsuan Lee, Yu-Ju Lin, Pauline Nguyen, Sam Trinh, Hansen Dang, Mindie H. Nguyen

**Affiliations:** 1 Division of Gastroenterology, University of Washington, Seattle, WA 98195 USA; 2 Department of Medicine, Stanford University Medical Center, Palo Alto, CA 94305 USA; 3 Division of Gastroenterology and Hepatology, Stanford University Medical Center, Palo Alto, CA 94305 USA; 4 Institute of Clinical Medicine, National Yang-Ming University, Taipei, Taiwan

## Abstract

The effect of metabolic syndrome on chronic liver diseases other than non-alcoholic fatty liver disease has not been fully elucidated. Our goal was to evaluate if metabolic syndrome increased the risk of liver-related complications, specifically hepatocellular carcinoma (HCC) and decompensation, in cirrhotic chronic hepatitis C (CHC) patients. We conducted a retrospective cohort study of 3503 consecutive cirrhotic CHC patients seen at Stanford University from 1997–2015. HCC developed in 238 patients (8-year incidence 21%) and hepatic decompensation in 448 patients (8-year incidence 61%). The incidence of HCC and decompensation increased with Hispanic ethnicity, diabetes, and number of metabolic risk factors. Multivariate Cox regression analysis demonstrated that, independent of HCV therapy and cure and other background risks, Hispanic ethnicity with ≥2 metabolic risk factors significantly increased the risk of HCC and hepatic decompensation. There was no interaction between Hispanic ethnicity and metabolic risk factors. All in all, metabolic risk factors significantly increase the risk of liver-related complications in cirrhotic CHC patients, especially HCC among Hispanics. As the prevalence of metabolic syndrome increases globally, targeted health interventions are needed to help curb the effects of metabolic syndrome in CHC patients.

## Introduction

The prevalence of metabolic syndrome has increased exponentially over the past 40 years^1,2^. According to the National Health and Nutrition Examination Survey (NHANES) data from 1999–2006, 68 million US adults were estimated to have metabolic syndrome^3^. Non-alcoholic fatty liver disease is often equated as the hepatic manifestation of metabolic syndrome. It has been linked to increased incidence of hepatic decompensation–including both cirrhosis and hepatocellular carcinoma (HCC)^4,5^.

Previous studies have shown an increased risk of liver-related mortality with obesity and other metabolic risk factors. However, less is known about the effect of metabolic risk factors on other causes of chronic liver diseases. Hepatitis C virus (HCV) is the second leading cause of chronic liver disease in the United States. It currently affects 80 million people worldwide^6^ and is the leading cause of liver transplantation in the United States^7^. Previous papers have demonstrated an increased risk of primary liver cancer in hepatitis C patient with excess body weight^8,9^ and diabetes^10,11^. However, to our knowledge, there is no large-scale analysis studying the impact of metabolic syndrome on the natural history of chronic hepatitis C infection among patients with cirrhosis.

Therefore, the aim of this study was to evaluate the impact of metabolic syndrome on liver-related complications in a large ethnically diverse cohort of chronic hepatitis C (CHC) patients with cirrhosis, specifically evaluating for risk of hepatic decompensation and HCC.

## Results

### Baseline characteristics

There were a total of 3503 patients with HCV cirrhosis included in the study. Table 1 shows the baseline clinical characteristics. The majority of patients (62%) were male and the average age was 54 years (SD ± 9.7). Half of the patients (50%) were white, 9% Asian, 22% Hispanic, and 19% other. A total of 14% of patients had metabolic syndrome per National Cholesterol Education Panel Adult Treatment Panel III (NCEP ATP III) definition. The average BMI was 29 (SD ± 6.8), 27% of patients had diabetes, 42% had hypertension, and 12% had hyperlipidemia. Almost half of the patients (46%) had a history of some alcohol use. The majority of patients (71%) had HCV genotype 1 disease. Forty percent of patients received anti-viral therapy, of which 33% achieved sustained virologic response (SVR). Of the treated patients, 44% received interferon-based therapy, while 56% received direct-acting antiviral therapy. The median follow-up time was 27 months (IQR 9–57 months) or 10,215 person-years. Per study criteria, no patients had HCC at baseline. Almost half (49%) of the study cohort had hepatic decompensation at the start of the study.

**Table 1:** Baseline clinical characteristics for 3503 patients with HCV cirrhosis.

	Overall (n = 3503)
Age	54.4 ± 9.7
Sex (male)	61.80%
Ethnicity	
White	50.40%
Hispanic	21.60%
Asian	8.80%
Other	19.20%
Metabolic risk factors	
Diabetes Type II	27.30%
Hypertension	41.60%
Hyperlipidemia	12.20%
Body Mass Index	29.0 ± 6.8
Metabolic syndrome	13.90%
Significant history alcohol use	46.30%
Child Pugh Score	
A	44.10%
BC	55.90%
Anti-viral therapy	40.10%
Achieved SVR	33.40%
Hepatocellular carcinoma (HCC)	
Baseline HCC	0%
Hepatic Decompensation	
Baseline decompensation	49.90%

Abbreviations: SVR, sustained virilogic response; HCC, hepatocellular carcinoma.

### Effect of metabolic risk factors on development of HCC

Table 2 shows baseline characteristics based on the development of HCC. Patients who developed HCC were older (average age 56.4 ± 9.4 vs 54.2 ± 9.7; *p* < 0.001) and more likely to be male (70.6% vs 61.1%; *p* = 0.004) compared to patients who did not develop HCC. Patients who developed HCC had higher prevalence of metabolic risk factors. They were more likely to have diabetes (40.8% vs 26.3%, *p* < 0.001), high blood pressure (56.7% vs 40.5%, *p* < 0.001), and metabolic syndrome (21.4% vs 13.3%, *p* < 0.001). They were also more likely to have more advanced liver disease with increased Child Pugh BC scores (73.5% vs 54.4%, *p* < 0.001). There was no difference in alcohol use between groups.

**Table 2:** Baseline clinical characteristics for 3503 patients with HCV cirrhosis by HCC status.

	No HCC	Developed HCC	*P-*value
	(n = 3265)	(n = 238)	
Age	54.2 ± 9.7	56.4 ± 9.4	<0.001
Sex (male)	61.10%	70.60%	0.004
Metabolic syndrome	13.30%	21.40%	<0.001
Diabetes Type II	26.30%	40.80%	<0.001
Hypertension	40.50%	56.70%	<0.001
Hyperlipidemia	12.10%	13.90%	0.420
Body Mass Index	29.0 ± 6.9	28.2 ± 5.5	0.110
Coronary Artery Disease	10.60%	19.80%	<0.001
Significant history alcohol use	46.30%	46.60%	0.930
Ethnicity			
White	50.30%	51.30%	<0.001
Asian	21.20%	27.70%	
Hispanic	8.50%	13.00%	
Other	20.00%	8.00%	
Child Pugh Score			
A	45.60%	26.50%	<0.001
BC	54.40%	73.50%	

Abbreviations: HCC, hepatocellular carcinoma; HCV, hepatitis C virus.

**Figure 1:**
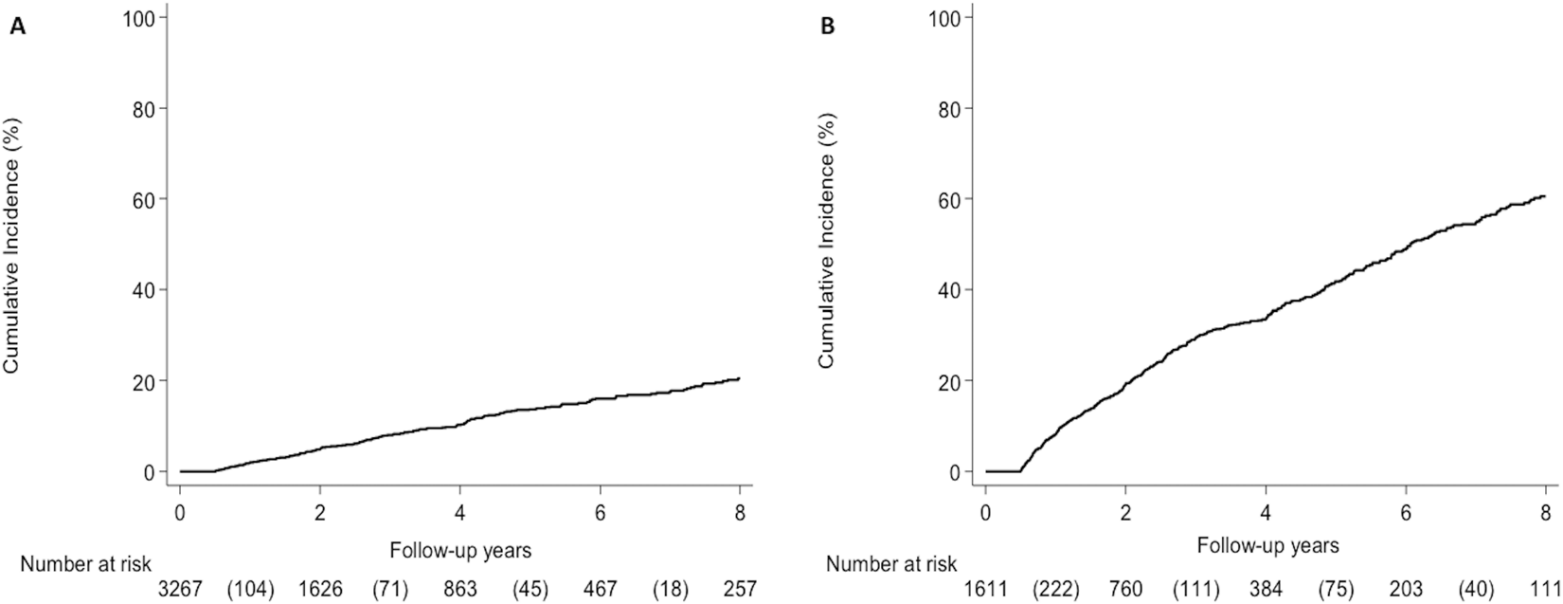
Development rate of (**A**) hepatocellular carcinoma and (**B**) hepatic decompensation.

**Figure 2:**
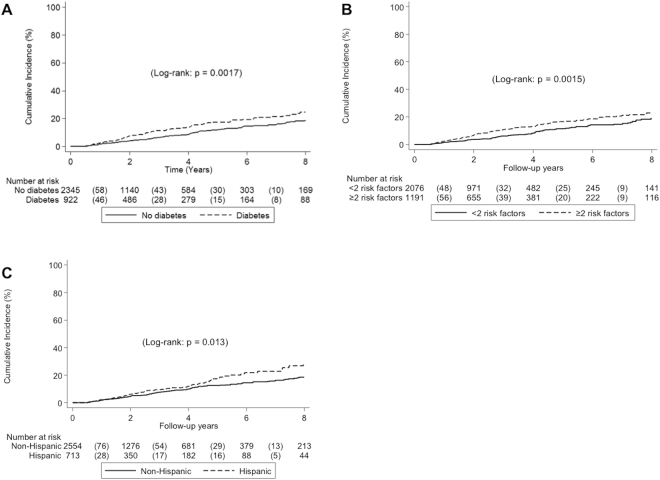
Incidence of hepatocellular carcinoma by (**A**) diabetes status (**B**) number of metabolic risk factors and (**C**) Hispanic ethnicity.

**Figure 3:**
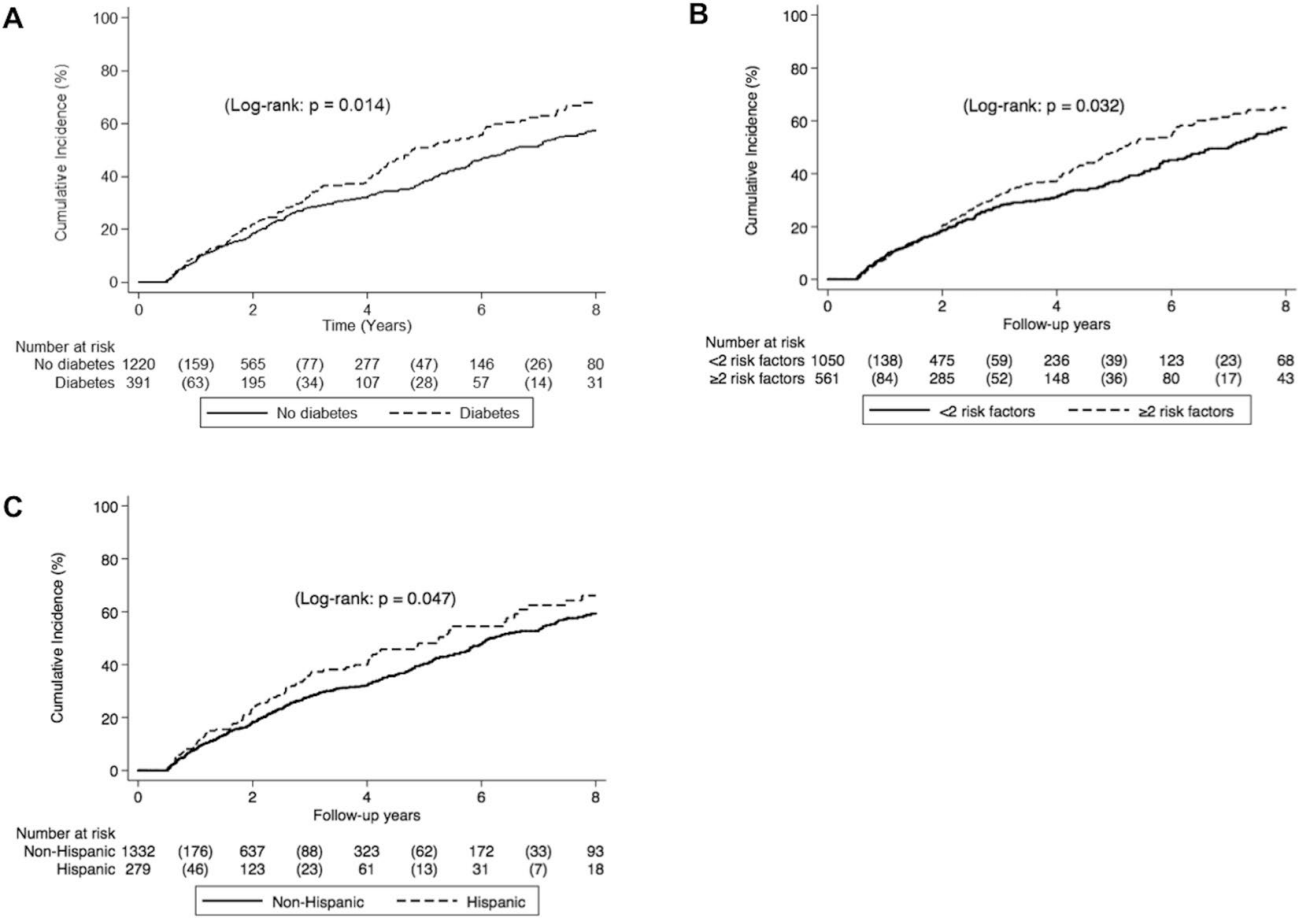
Incidence of hepatic decompensation by (**A**) diabetes status (**B**) number of metabolic risk factors and (**C**) Hispanic ethnicity.

The 8-year incidence of HCC was 20.5% or 2.66 100 person-years (Fig. 1A). HCC developed more frequently among patients with diabetes compared to patients without diabetes (Fig. 2A). The 8-year incidence for patients with diabetes was 24.5% (3.5 100 person-years) compared to 18.6% (2.29 100 person years) for patients without diabetes (*p* = 0.002). HCC also developed more frequently among patients with increased number of metabolic risk factors (Fig. 3B). The 8-year incidence of HCC for patients with 2 or more metabolic risk factors was 22.9% (3.35 100 person-years) versus 18.8% (2.18 100 person years) for patients with 0 or 1 metabolic risk factors (*p* = 0.002). There was no difference in HCC incidence by BMI or excess body weight. When we evaluated the incidence of HCC by ethnicity, Hispanic patients developed HCC more frequently than non-Hispanic patients (Fig. 3C). The 8-year incidence for Hispanic patients was 28.4% (3.49 100 person-years) compared to 18.4% (2.44 100 person-years) for non-Hispanic patients (*p* = 0.013).

Cox regression analysis showed that older age, male sex, Child Pugh score, lack of SVR after hepatitis C treatment, and Hispanic patients with greater than two metabolic risk factors significantly increased risk of HCC (Table 3). Multiplicative interaction testing was subsequently performed and did not show an interaction between Hispanic ethnicity and metabolic risk factors (*p* = 0.44). Alcohol use was not included in the cox regression analysis as it was not a significant predictor for HCC on univariate analysis (HR = 0.96, CI 0.75–1.24; *p* = 0.76).

**Table 3:** Predictive factors for the development of HCC.

	Unadjusted HR (95% CI)	*P* value	Adjusted HR (95% CI)	*P* value
Age (5-Year intervals)	1.19 (1.12–1.27)	<0.001	1.29 (1.18–1.40)	<0.001
Male sex	1.62 (1.23–2.15)	0.001	1.64 (1.15–2.34)	0.006
Ethnicity				
Non-Hispanic < 2 MRF	Referent	Referent	Referent	Referent
Hispanic < 2 MRF	1.14 (0.73–1.79)	0.570	1.42 (0.80–2.54)	0.230
Non-Hispanic ≥ 2 MRF	1.35 (0.99–1.82)	0.052	1.00 (0.68–1.48)	0.990
Hispanic ≥ 2 MRF	2.17 (1.50–3.13)	<0.001	1.89 (1.20–2.97)	0.006
CTP score (continuous)	1.16 (1.08–1.25)	<0.001	1.14 (1.06–1.24)	0.001
SVR status				
SVR	Referent	Referent	Referent	Referent
No SVR	2.88 (1.48–5.60)	0.002	2.53 (1.18–5.42)	0.017

Multiplicative interaction *P*-value: 0.44.

Abbreviations: CTP, Child- Turcotte-Pugh; HCC, hepatocellular carcinoma; MRF, metabolic risk factor; SVR, sustained virologic response.

### Effect of metabolic risk factors on hepatic decompensation

Of the 1775 patients who did not have hepatic decompensation at baseline, 448 developed hepatic decompensation during the study period. Patients who had hepatic decompensation at baseline or developed hepatic decompensation were slightly younger than patients who did not develop hepatic decompensation (average age 53.6 ± 9.6 and 53.8 ± 8.9 respectively vs 55.6 ± 10.0; *p* < 0.001). Similar to the patients who developed hepatic decompensation, patients who had hepatic decompensation at baseline or developed hepatic decompensation were more likely to have diabetes (31.3% and 31.0% respectively vs 20.8%; *p* < 0.001), high blood pressure (41.8% and 46.9% respectively vs 39.5%; *p* = 0.022), and metabolic syndrome (14.1% and 16.7% respectively vs 12.7%; *p* = 0.092). There was increased alcohol use in patients with baseline hepatic decompensation or who developed hepatic decompensation compared to patients who did not have hepatic decompensation (52.4% and 46.8%, vs 38.3%; *p* =  < 0.001). As expected, patients with baseline hepatic decompensation or who developed hepatic decompensation, also had more advanced liver disease with higher percentage of Child Pugh BC disease (79.1% and 65.1% respectively vs 19.5%; *p* < 0.001).

The 8-year incidence of hepatic decompensation was 60.5% or 10.72 100 person-years (Fig. 1B). Patients with diabetes had significantly higher incidence of hepatic decompensation compared to patients without diabetes (Fig. 3A). The 8-year incidence for patients with diabetes was 67.8% (12.93 100 person-years) compared to 57.4% (9.96 100 person-years) for patients without diabetes (*p* = 0.014). Similarly, patients with two or more metabolic risk factors had higher incidence of hepatic decompensation than patients with 0 to 1 metabolic risk factors (Fig. 3B). The 8-year incidence of hepatic decompensation for patients with 2 or more metabolic risk factors was 64.9% (12.22 100 person-years) versus 57.5% (9.84 100 person-years) for patients with 0 or 1 metabolic risk factors (*p* = 0.032). There was no difference in incidence of hepatic decompensation by BMI or excess body weight. When we evaluated the incidence of hepatic decompensation by ethnicity, Hispanic patients developed hepatic decompensation more frequently than non-Hispanic patients (Fig. 3C). The 8-year incidence for Hispanic patients was 66.1% (12.89 100 person-years) compared to 59.3% (10.29 100 person-years) for non-Hispanic patients (*p* = 0.047).

The factors associated with the development of hepatic decompensation are shown in Supplemental Table 1. Multivariate analysis demonstrated that older age, increased Child Pugh score, and Hispanic ethnicity with greater than two metabolic risk factors significantly increased risk of hepatic decompensation. Multiplicative interaction testing was subsequently performed and did not show an interaction between Hispanic ethnicity and metabolic risk factors (*p* = 0.58).

## Discussion

To our knowledge this is the first large cohort study in the United States to evaluate the effect of metabolic syndrome on liver-related complications in CHC patients. In our cohort of 3503 cirrhotic CHC patients, 238 patients (6.7%) developed HCC. Half (49%) of patients had baseline decompensation; and of the other half (51%) who did not have baseline hepatic decompensation, 448 patients (25.2%) developed hepatic decompensation. The patients who developed liver-related complications were more likely to have hypertension, diabetes, and metabolic syndrome or metabolic risk factors. In addition, the incidence of liver-related complications was increased in patients with diabetes or metabolic risk factors. The incidence of liver-related complications was also increased in Hispanic compared to non-Hispanic patients. Multivariate analysis demonstrated that, following adjustment for various background risks including HCV therapy and treatment response, Hispanic patients with two or more metabolic risk factors had 89% higher risk of developing HCC and 60% higher risk of developing hepatic decompensation.

There has been a growing body of evidence that metabolic risk factors such as diabetes and obesity are associated with progression of chronic liver disease, especially in HCV infection which has independently been connected with insulin resistance^12,13^, steatosis^14^, and lipogenesis^15^. The largest study to date by Arase *et al*. in Japan found a 1.7-fold enhancement in the development of HCC in hepatitis C patients with diabetes^10^. Similarly, a meta-analysis by Dyal *et al*. evaluated six other studies (all in Asia) in addition to the study by Arase and colleagues, and demonstrated that diabetes was significantly associated with increased risk of HCC in CHC patients (HR 1.73)^16^. However, even this association remains contested. Two of the papers evaluated by Dyal and colleagues only saw a non-significant trend toward higher HCC risk associated with diabetes and a study by Yang *et al*. evaluating data from Mayo Clinic and the hepatitis C antiviral long-term treatment against cirrhosis (HALT-C) trial found no association between diabetes and HCC in patients with HCV cirrhosis^17^.

In our large cohort study in the United States, we found a significant association between diabetes and the development of liver-related complications. The incidence of hepatic decompensation and HCC were both significantly increased in these patients. Insulin resistance, which is thought to create a pro-inflammatory state promoting hepatocyte injury and proliferation of collagen production, may be driving this association^18^.

Previous studies have shown positive associations between excess body weight and risk of HCC in HCV patients. In a large meta-analysis by Chen and colleagues, patients with HCV infection were found to have a higher risk of primary liver cancer with excess body weight (BMI > 25) (Summary RR 2.15, 95% CI 1.50–3.90)^8^. We were not able to reproduce these results despite using ethnically specific BMI cut-offs. Neither excess body weight nor obesity was significantly associated with increased risk of hepatic decompensation or HCC. In our study, BMI was measured at the date of cirrhosis diagnosis. It may be that we were evaluating people too late in their disease course and that their weight at the time of cirrhosis was no longer representative of their baseline weight either secondary to weight loss from malnutrition or weight gain in the setting of volume overload which is often associated with end-stage liver disease.

The prevalence of metabolic syndrome was 14% in our study. This is similar to another study which found 13.2% of patients with HCV also had metabolic syndrome^19^. While the prevalence of HCV infection has stabilized, the prevalence of chronic hepatitis C with metabolic syndrome or DMII has continued to increase with the increasing obesity epidemic. According to data from the NHANES registry, 45% of patients with HCV had concurrent metabolic syndrome from 2011–2012^20^. The increased prevalence observed in the NHANES study is likely secondary to the significantly increased number of African Americans (34% vs 4.3%) and decreased number of Asian Americans in the NHANES data compared to our patient population. In the NHANES dataset, the highest rates of metabolic syndrome were observed in African Americans.

While there is a large amount of data linking diabetes and obesity to liver-related complications in HCV, there is less data evaluating metabolic syndrome as a whole. Stepanova and colleagues attempted to fill in this gap by examining the association between metabolic syndrome with over-all and liver-related mortality in four different types of chronic liver disease. They evaluated 264 patients with chronic hepatitis C and found metabolic syndrome to be an independent predictor of liver-related (3.38, 95% CI 1.52–6.66) though not overall mortality (adjusted HR 1.67, 95% CI 0.63–4.45)^21^. While we did not evaluate mortality, our study supports these findings. Having greater than or equal to two metabolic risk factors was significantly associated with increased risk of HCC and hepatic decompensation.

Although we did not observe a significant interaction between metabolic syndrome and Hispanic ethnicity, Hispanic patients with increased metabolic risk factors had a significantly higher risk of developing hepatic decompensation and HCC. Similar racial differences were described by El-Serag and colleagues^22^. They evaluated 149,407 patients with active HCV viremia in the Veterans Administration HCV clinical case registry and found that Hispanic patients with hepatitis C had the highest annual incidence rates of cirrhosis and HCC. We were able to expand on the results of this study by analyzing data from both male and female patients, incorporating metabolic risk factors other than diabetes, and evaluating for possible interaction between Hispanic ethnicity and metabolic risk factors.

Our study had some limitations. We had increased number of Asian patients and decreased number of black patients thus making generalizability to the overall United States population difficult. The study period also spanned a time period with vast advances in hepatitis C treatment in the last few years that we could not account for in the study due to lack of long-term follow-up for most of the patients who were treated with newer antiviral agents. However, the finding on the effect of Hispanic ethnicity (n = 756) and increased metabolic risk factors on liver disease outcomes was independent of SVR status. Other limitations included our reliance on ICD-9 codes as surrogates for fasting blood glucose levels, lipid levels, and blood pressure measurements as well as our lack of waist circumference data. Though we attempted to account for the latter using an established formula to calculate waist circumference from BMI, the BMI calculation is only valid for Caucasians, African Americans and possibly Hispanics^23^.

The strengths of our study included its cohort study design with long-term follow up, its large study patient population including a large Hispanic population who were consecutively enrolled from all departments at our medical center and the breadth of data on metabolic risk factors, hepatic decompensation, and HCC derived from structured individual patient chart review using a case report form designed for this study.

In summary, this study shows that patients with CHC cirrhosis and super-imposed metabolic syndrome have increased risk of liver-related complications including both hepatic decompensation and HCC. Hispanic patients with two or more metabolic risks are at especially increased risk of developing liver-related complications. As the prevalence of obesity and metabolic syndrome increase across the world, targeted health interventions will be needed to help curb the effects of metabolic syndrome in chronic hepatitis C patients.

## Patients and Methods

### Study Design and Patient Population

This is a retrospective cohort study of 3503 consecutive patients with Hepatitis C cirrhosis seen at Stanford University Medical Center from January 1997 to June 2015 (Supplemental Fig. 1). Patients were identified via ICD-9 diagnosis query for HCV (ICD-9 code 70.41, 70.51, 70.44, 70.54, 70.70, 70.71, v02.62) and subsequently confirmed by individual chart review to have chronic hepatitis C and cirrhosis (criteria as below). Patients were excluded if they had HCC at baseline or within the first six months.

### Clinical variables

Clinical data including laboratory values, pathology results and imaging results were reviewed and abstracted via individual record review using a case report form. Individual medical records were reviewed to confirm HCV diagnosis either by positive hepatitis C antibody (anti-HCV), HCV RNA, or documented history of HCV infection in physician clinical notes. Cirrhosis was determined by pathological evidence of stage 4 fibrosis, noninvasive testing consistent with stage 4 fibrosis, clinical evidence of portal hypertension (platelet count < 120,000/μL or splenomegaly, ascites, or gastroesophageal varices on imaging), or prior hepatic decompensation (hepatic encephalopathy, ascites, variceal gastrointestinal bleeding). Significant history of alcohol use was defined as either alcohol consumption of >21 standard drinks per week in men or > 14 standard drinks per week in women or the documented history of alcoholic cirrhosis in physician clinical notes. There was no set number of radiological or laboratory testing that patients’ had to undergo.

### Definition of excess body weight and metabolic risk factors

Per World Health Organization regional guidelines, we defined excess body weight as body mass index (BMI) > 23 kg/m^2^ for Asian and BMI > 25 kg/m^2^ for non-Asian patients. Obesity was defined as BMI > 25 kg/m^2^ for Asian and BMI > 30 kg/m^2^ for non-Asian patients^24,25^.

Metabolic syndrome was defined using NCEP ATP III guidelines^26^. According to these guidelines, metabolic syndrome is present if three or more of the following five criteria are met: waist circumference over 40 inches for men or 35 inches for women, blood pressure over 130/85 mmHg, fasting triglyceride level over 150 mg/dl, fasting high-density lipoprotein cholesterol level less than 40 mg/dl for men or 50 mg/dl for women and fasting blood sugar over 100 mg/dl. We did not have data on blood pressure measurements, fasting triglycerides, and fasting blood sugar and subsequently used ICD-9 diagnosis query for hypertension (ICD-9 code 401, 401.1, 409.1), hyperlipidemia (ICD-9 code 272.4), and diabetes ICD-9 code 249*) respectively in their stead. Waist circumference was estimated based on gender using BMI, age, and ethnicity (African American, Caucasian and Hispanic) as calculated per Bozeman *et al*. formula^23^.

### Outcomes

We measured two primary liver-related outcomes: development of HCC and hepatic decompensation. HCC diagnosis was confirmed via cytologic or pathologic diagnosis or based on noninvasive criteria by the American Association for the Study of Liver Diseases (elevated AFP and characteristic imaging)^27^. Hepatic decompensation was defined as evidence of jaundice, ascites, hepatic encephalopathy, or variceal bleed.

### Statistical analysis

Descriptive statistics were reported as proportion (%) for categorical variables, and mean ± standard deviation (SD) or median (and range) for continuous variables. The normally distributed continuous variables in different groups were compared by Student’s *t* test. The Wilcoxon rank-sum test was applied when continuous variables were not normally distributed. The chi-squared test was used to compare the categorical variables of different groups. Kaplan-Meier analyses were used to depict the curves of incidence for decompensated liver cirrhosis or hepatocellular carcinoma by ethnicity and the presence of diabetes. The differences of incidence of these interested outcomes were compared by log-rank tests. Patients were censored at death or their last date of follow-up at Stanford. The date of HCC or hepatic decompensation was used to determine incidence of the respective condition. The Cox proportional hazards model was used to estimate the hazard ratio and 95% confidence interval relating risk factors (demographic, comorbid conditions, and disease severity) to development of hepatic decompensation and HCC. Multiplicative interaction between metabolic risk factors and Hispanic ethnicity were assessed by adding a product term for Hispanic ethnicity multiplied by number of metabolic risk factors and evaluated using multivariate cox’s regression analyses. Statistical significance was defined as a 2-tailed P value < 0.05. All statistical analyses were performed using Stata 11.0 (Stata Corporation, College Station, TX). This study was approved by the Institutional Review Board at Stanford University (Stanford, CA). All research was performed in accordance with relevant guidelines and regulations. Informed consent was obtained from all participants and/or their legal guardians.

### Data availability

The datasets generated during/or analyzed during the current study are available from the corresponding author on reasonable request.

## Electronic supplementary material

Supplemental Information
