# Supplementary material for: Higher risk of hepatocellular carcinoma in Hispanic patients with hepatitis C cirrhosis and metabolic risk factors
Source: Sci Rep. 2018 May 8;8:7164. doi: 10.1038/s41598-018-25533-2 (PMC5940826; doi:10.1038/s41598-018-25533-2)

**Higher risk of hepatocellular carcinoma in Hispanic patients with hepatitis C cirrhosis and metabolic risk factors.**

Alina Wong^1,2^, An Le^3^, Mei-Hsuan Lee^4^, Yu-Ju Lin^4^, Pauline Nguyen^3^, Sam Trinh ^3^, Hansen Dang^3^, Mindie H. Nguyen^3^

^1^ Division of Gastroenterology, University of Washington, Seattle, WA 98103

^2^ Department of Medicine, Stanford University Medical Center, Palo Alto, CA 94305

^3^ Division of Gastroenterology and Hepatology, Stanford University Medical Center, Palo Alto, CA 94305

^4^ Institute of Clinical Medicine, National Yang-Ming University, Taipei, Taiwan

**Supplemental table 1. Predictive factors for the development of hepatic decompensation**

|  | Unadjusted HR (95% CI) | *P* value | Adjusted HR  (95% CI) | *P* value |
| --- | --- | --- | --- | --- |
| Age (5-Year intervals) | 1.01 (0.96 – 1.06) | 0.720 | 1.11 (1.05 – 1.19) | 0.001 |
| Male sex | 0.98 (0.81 – 1.18) | 0.800 | 0.79 (0.61 – 1.02) | 0.066 |
| Ethnicity |  |  |  |  |
| Non-Hispanic <2 MRF | Referent | Referent | Referent | Referent |
| Hispanic <2 MRF | 1.20 (0.88 – 1.64) | 0.250 | 1.11 (0.73 – 1.69) | 0.630 |
| Non-Hispanic ≥2 MRF | 1.20 (0.97 – 1.48) | 0.090 | 1.22 (0.92 – 1.63) | 0.170 |
| Hispanic ≥2 MRF | 1.62 (1.15 – 2.27) | 0.005 | 1.60 (1.06 – 2.41) | 0.025 |
| CTP score (continuous) | 1.36 (1.29 – 1.43) | <0.001 | 1.39 (1.31 – 1.47) | <0.001 |
| SVR status |  |  |  |  |
| SVR | Referent | Referent | Referent | Referent |
| No SVR | 2.23 (1.42 – 3.49) | <0.001 | 1.25 (0.74 – 2.10) | 0.400 |

Multiplicative interaction *P*-value: 0.58

Abbreviations: CTP, Child- Turcotte-Pugh; MRF, metabolic risk factor; SVR, sustained virologic response


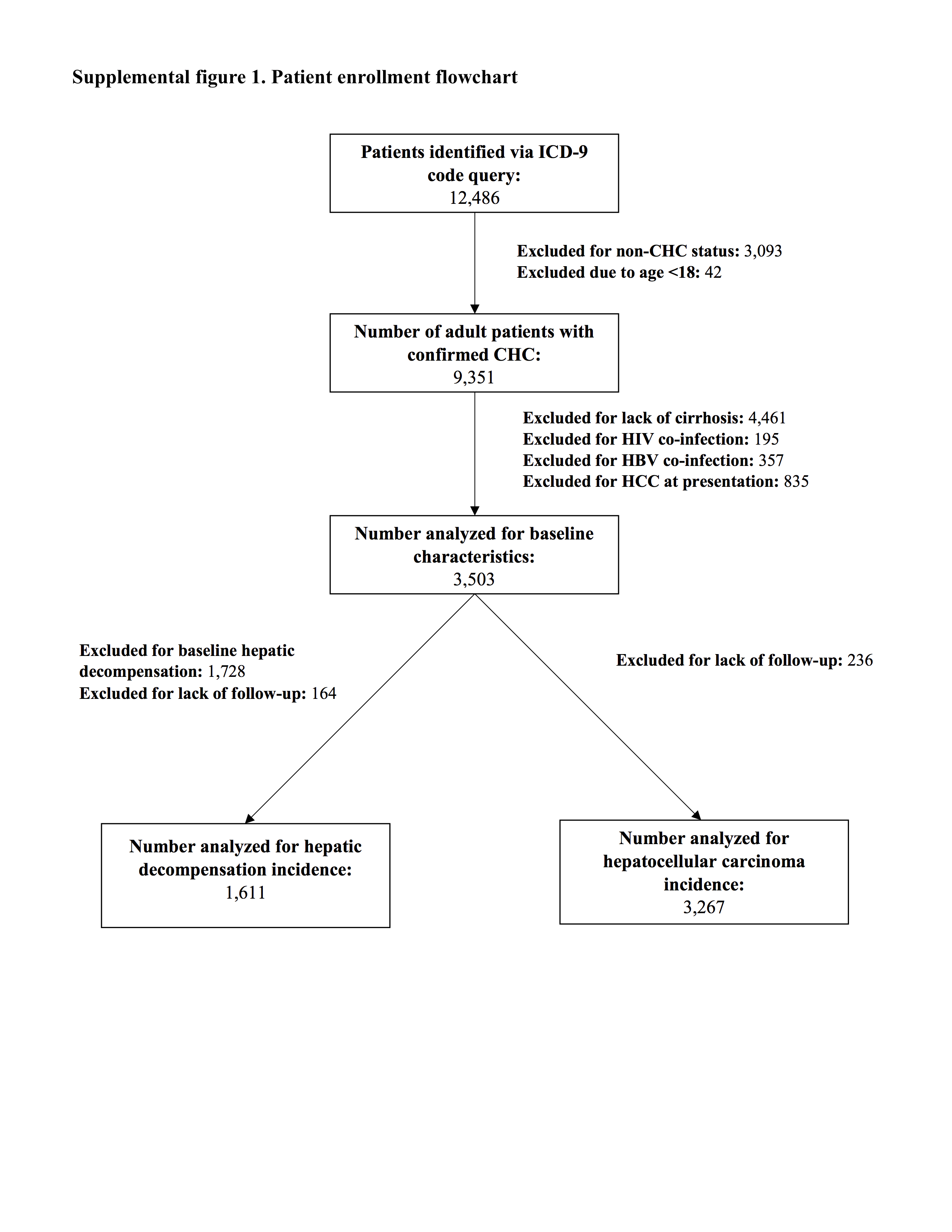

Supplement: Supplementary file 1 — Supplemental Information [file 41598_2018_25533_MOESM1_ESM.docx]
